# Fibromuscular dysplasia: what the radiologist should know: a pictorial review

**DOI:** 10.1007/s13244-015-0382-4

**Published:** 2015-04-30

**Authors:** L. Varennes, F. Tahon, A. Kastler, S. Grand, F. Thony, J. P. Baguet, O. Detante, E. Touzé, A. Krainik

**Affiliations:** 1 Department of Neuroradiology and MRI, University Hospital of Grenoble, CS 10217-38043, Grenoble Cedex 09, France; 2 Department of Radiology, University Hospital of Grenoble, CS 10217-38043, Grenoble Cedex 09, France; 3 Department of Cardiology, Mutualist Hospital Group of Grenoble, 38028 Grenoble cedex 1, France; 4 Department of Neurology, University Hospital of Grenoble, CS 10217-38043, Grenoble Cedex 09, France; 5 Department of Neurology, University Caen Basse Normandie, Inserm U919, CHU Côte de Nacre, Caen, France

**Keywords:** Fibromuscular dysplasia, Radiology, Arteries, Aneurysm

## Abstract

**Abstract:**

Fibromuscular dysplasia (FMD) is an idiopathic, segmentary, non-inflammatory and non-atherosclerotic disease that can affect all layers of both small- and medium-calibre arteries. The prevalence of FMD is estimated between 4 and 6 % in the renal arteries and between 0.3 and 3 % in the cervico-encephalic arteries. FMD most frequently affects the renal, carotid and vertebral arteries, but it can theoretically affect any artery. Radiologists play an important role in the diagnosis of FMD, and good knowledge of FMD’s signs will certainly help reduce the delay between the first symptoms and diagnosis. The common string-of-beads aspect is well known, but less common presentations also have to be considered. These less common imaging findings include vascular loops, fusiform vascular ectasia, arterial dissection, aneurysm and subarachnoid haemorrhage. These radiologic presentations should be known by radiologists in order to diagnose possible FMD, particularly when present in young females or when associated with personal or familial hypertension, to reduce the delay between the onset of the first symptom and the final diagnosis. The patients have to be referred to specialised FMD centres for dedicated management.

***Teaching Points*:**

• *Fibromuscular dysplasia is not a rare disease.*

• *Radiologists should recognise less common presentations to orient specific management.*

• *Vascular loops, fusiform vascular ectasia and a “string-of-beads” aspect are typical presentations.*

• *Arterial dissection, aneurysm and subarachnoid haemorrhage are less typical radiologic presentations.*

## Introduction

Fibromuscular dysplasia (FMD) is an idiopathic, segmentary, non-inflammatory and non-atherosclerotic disease that can affect all layers of both small- and medium-calibre arteries. Although not known, the FMD prevalence in the general population is considered to be low by several authors [1]. The cause and pathophysiology of FMD are still unknown [2, 3]. Clinical manifestations of FMD are primarily dependent on the vessels that are involved. FMD most frequently affects the renal, carotid and vertebral arteries, but can be found in all territories [3]. It is a potentially serious disease, especially in the cervico-encephalic location, where it can lead to severe stenosis, hypoperfusion, aneurysm, subarachnoid haemorrhage, dissection or arterial occlusion [3–5].

FMD is often not considered in the differential diagnosis because radiological aspects can be difficult to diagnose and the symptoms reported are not specific. Therefore, the mean delay from symptoms to diagnosis has been reported to be up to 4.1 years [6].

Radiologists play an important role in the diagnosis of FMD, and a good knowledge of FMD’s presentations should help to reduce the delay between the first symptoms and final diagnosis. The objective of this review is therefore to raise radiologists’ awareness of FMD’s epidemiology, pathophysiology, clinical presentation, typical and atypical radiological aspects and possible complications.

## Epidemiology

The prevalence of FMD is estimated between 4 and 6 % in the renal arteries and between 0.3 and 3 % in the cervico-encephalic arteries [2, 7–9]. However, most data stem from series of consecutive angiograms performed in symptomatic patients and therefore cannot be considered representative. Several authors believe that these prevalence values are underestimated because FMD is frequently asymptomatic [10–12]. In a review combining the results of 4 studies including 3,181 asymptomatic patients who had undergone a renal angiography before kidney transplant donation, 4.4 % (139 subjects) presented FMD lesions [1]. In another study among 20,244 consecutive autopsies performed at the Mayo Clinic over a 25-year period, only 4 subjects had cervical FMD (0.02 %) [13].

FMD is most frequently revealed in the kidneys in young patients with resistant hypertension secondary to fibrodysplastic renal artery stenosis (RAS). FMD is found in about 1 % of hypertensive patients and represents the second leading cause of renovascular hypertension after atherosclerotic disease. As for cervico-encephalic FMD, the symptoms are not specific.

In 2012, Olin et al. [6] included 447 cases from the FMD registry from nine states in the USA, the largest number of patients included in any study to date. The most frequently described symptoms or conditions were hypertension (63.8 %), headaches (52.4 %), pulsatile tinnitus (27.5 %), dizziness (26 %) and neck pain (22 %). This study showed a clear female prevalence (91 %), and the mean age at diagnosis was 51.9 years. In 6 % of cases, FMD was discovered randomly. Affected arteries were mainly renal arteries (79.7 %), extracranial carotid (74.4 %) and vertebral (36.6 %) arteries, mesenteric arteries (26 %) and intracranial carotid arteries (17 %). Sixty-five per cent of patients with renal FMD lesions also had cervico-encephalic artery lesions, and vice versa.

Several studies have reported a lower frequency of FMD lesions in the carotid arteries compared with renal arteries [1, 3, 14–16]. In 1982, Mettinger et al. found 58 % renal artery involvement versus 32 % carotid artery involvement based on a systematic review of 36 studies, including 1,197 patients with FMD [9]. This difference can be explained as FMD is more frequently symptomatic in renal arteries through the onset of hypertension [3], which also tends to increase its prevalence. Moreover, at the time of this study, multi-detector computed tomography (CT) was not as available a diagnostic tool as it is today. Indeed, easy access to computed tomographic angiography (CTA) and better knowledge of cervico-encephalic FMD, which is now looked for systematically in patients with renal artery lesions, may explain the differences in cervico-encephalic FMD lesion prevalence found in more recent studies [6].

## Pathophysiology

FMD’s pathophysiology remains unclear. Some authors suggest a role played by estrogenic impregnation given the female predominance. The predominance of right renal artery lesions suggests a mechanical component because the right kidney is more mobile than the left one; the mechanism may involve compression of the vasa vasorum leading to ischaemia. The vasa vasorum are small blood vessels that wind along arteries whose calibres are larger than 1 mm. They feed the adventitia and the external two-thirds of the media. There are relatively few in the carotid and renal arteries, possibly a feature of FMD [13]. Smoking could also be a risk factor. Autosomal dominant transmission with incomplete penetrance and variable expressivity is suggested in 6–10 % familial types; no specific gene has been identified, though [17]. Moreover, a study performed over 9 years in 106 patients who had repeated angiograms showed either a progression or a stabilisation of FMD lesions but no lesion regression was seen [9].

## Typical and variant aspects

### Typical aspects

#### Classifications

The first case of histologically proven carotid FMD was published by Connett and Lansche in 1965 [18]. Later, a classification of FMD based on renal artery lesions was described in 1971 by Harrison and MacCormack [19], then revised by Stanley in 1996 [20]. This classification is based on the most affected arterial layer: the intimal type, medial type or subadventitial type. The authors report that several forms can co-exist in the same patient. These lesions were later described in carotid arteries in autopsies [3].

1. The intimal type corresponds to fibrous circumferential intimal thickening with proliferation of subendothelial connective tissue. The internal elastic lamina is always preserved, and the media and adventitia are normal. This form is mainly found in children, rarely in adults (5 %). It appears as focal truncal stenosis (Fig. 1).

**Fig. 1:**
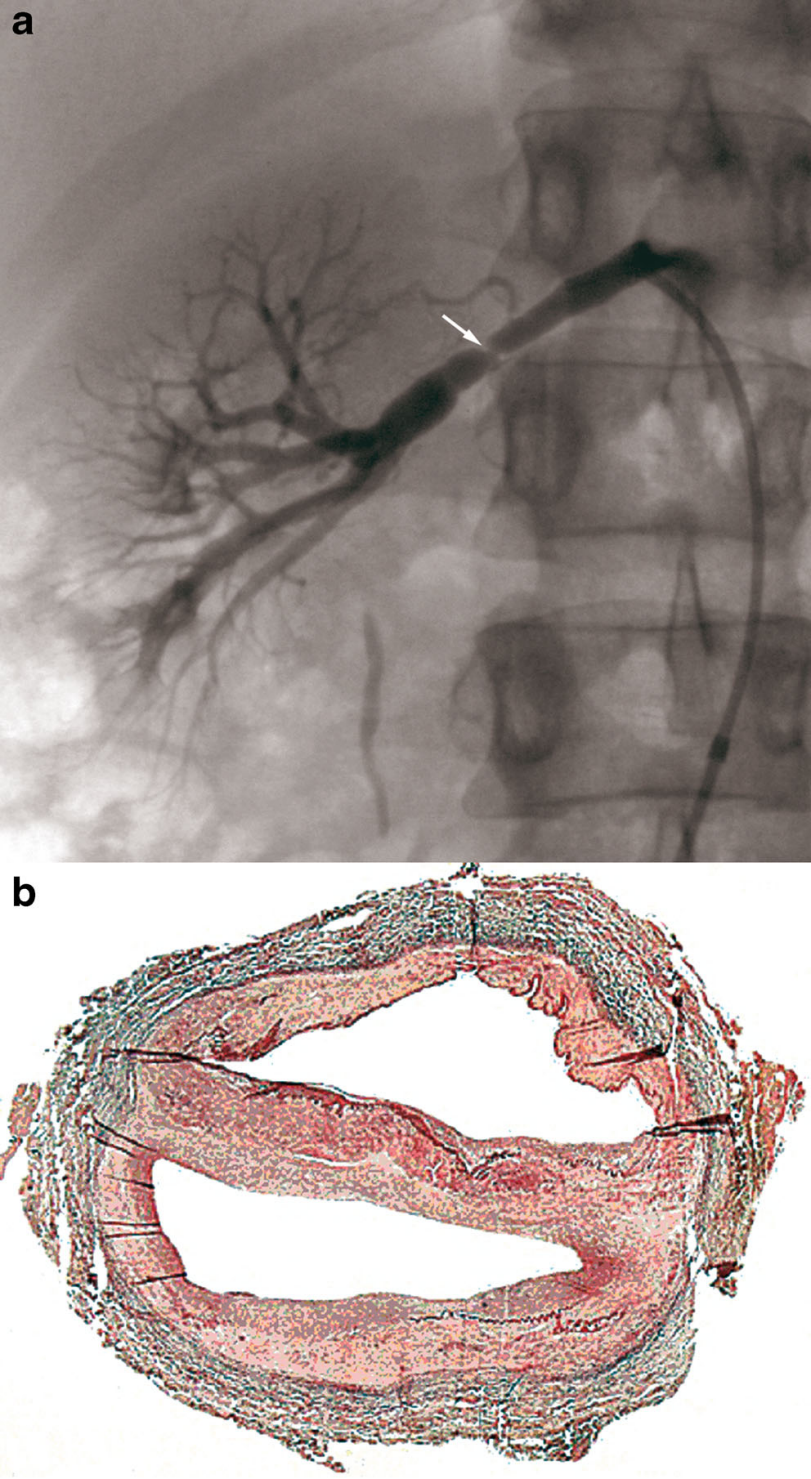
Example of intimal FMD lesions. **a** Renal angiogram showing short focal stenosis (*white arrow*) of the right renal artery, in favour of intimal FMD. **b** Colour histological slide of intimal FMD showing circumferential focal thickening. Media and adventitia are normal
2. The medial type is the most frequent (60–70 %) and corresponds to a rarefaction of smooth media muscle cells replaced by fibrosis. The intima, internal elastic lamina and adventitia are normal. It appears as a succession of dilatations and multifocal stenoses with a characteristic string-of-beads aspect. It is mainly discovered in females aged 30–50 years (Fig. 2).

**Fig. 2:**
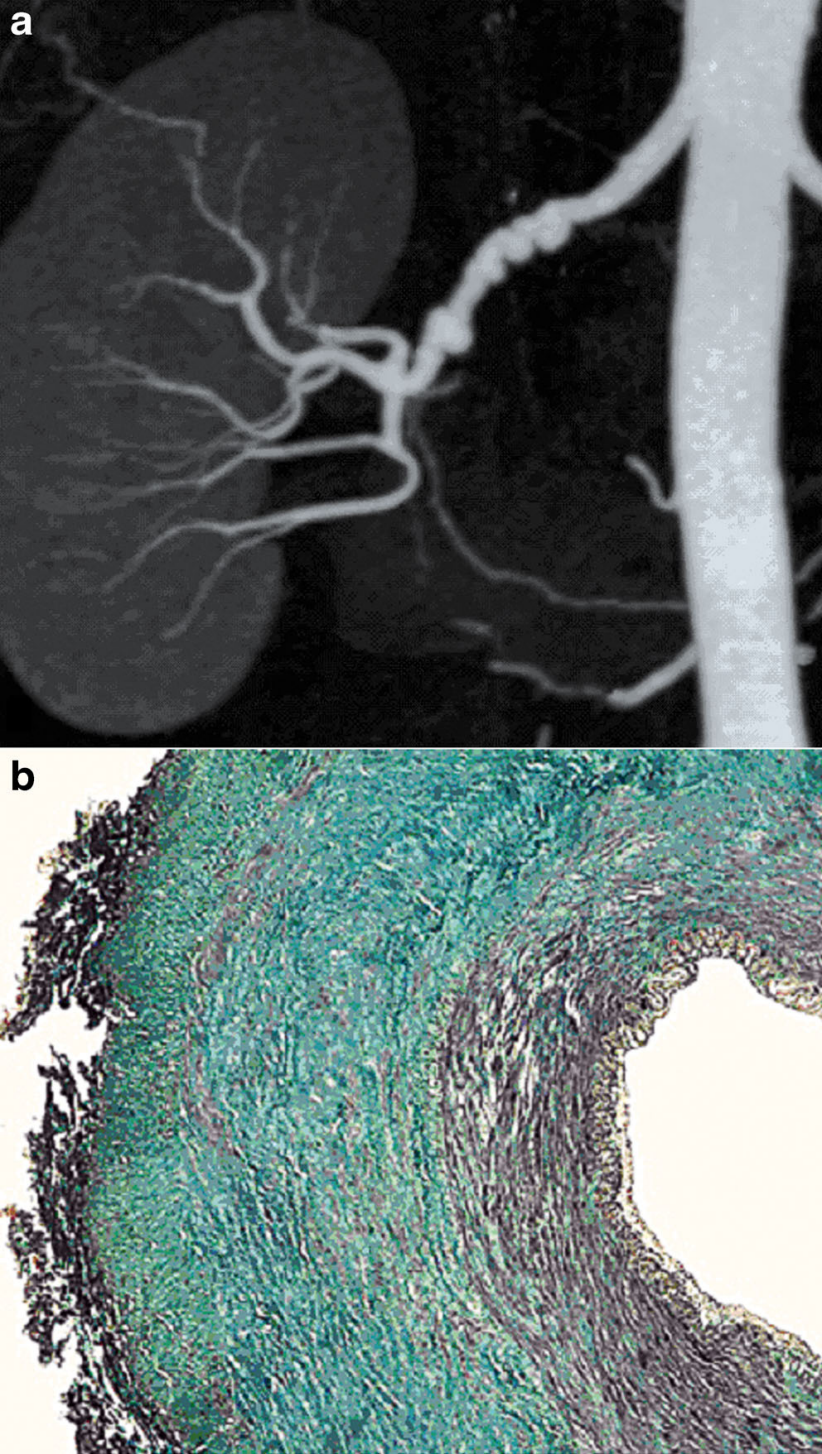
Example of medial FMD lesions. **a** Renal CTA with coronal plane MIP reconstruction, showing the typical “string-of-beads” aspect of the right renal artery, in favour of medial FMD. **b** Colour histological slide of medial FMD showing extensive medial fibrosis. Intima and adventitia are normal
3. The perimedial or subadventitial type (10–20 % of cases) corresponds to excessive elastic tissue in the external area of the media. Its aspect is close to the medial type with fewer dilatations that do not exceed the diameter of the normal artery. Stenosis often appears tubular (Fig. 3).

**Fig. 3:**
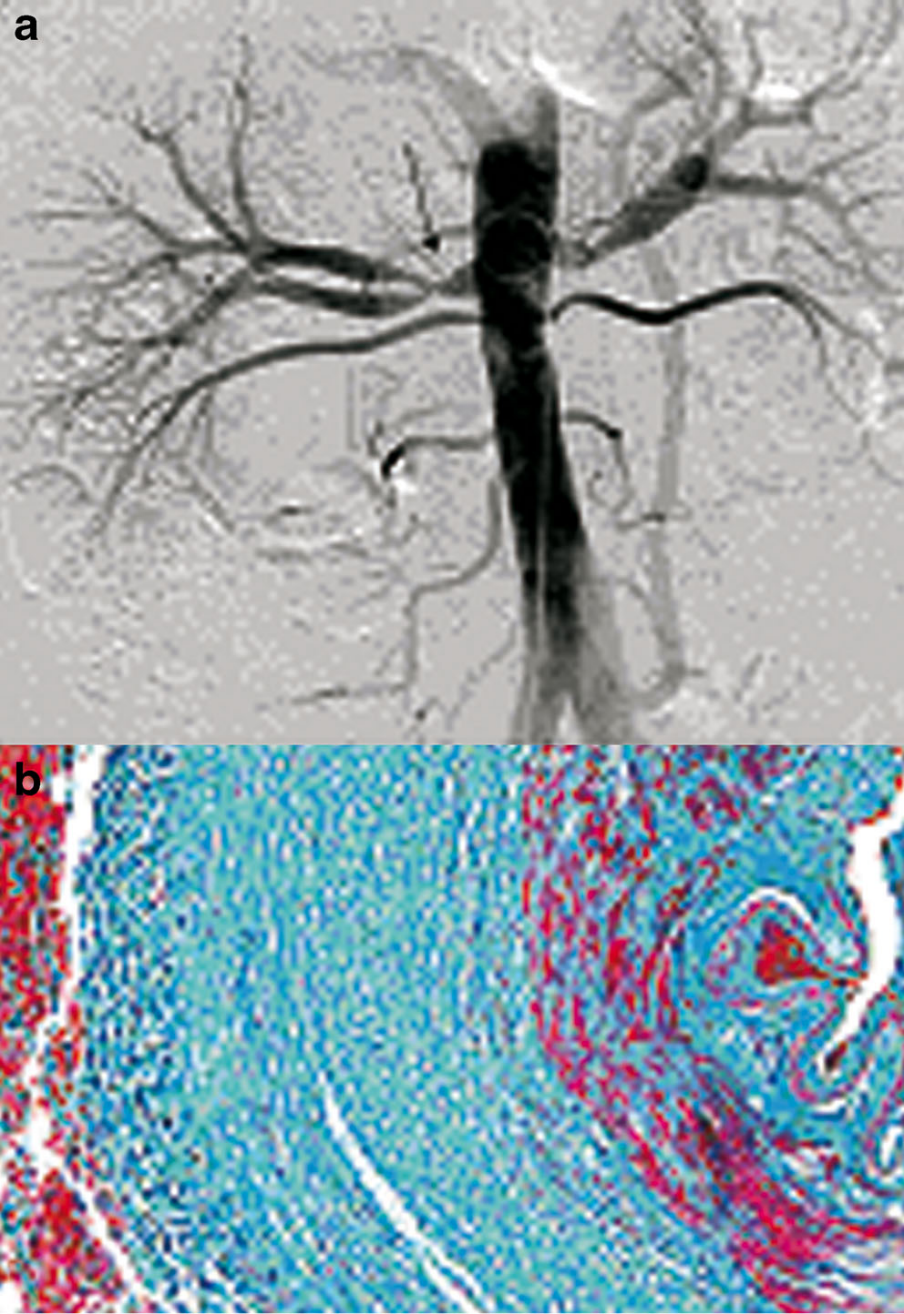
Example of perimedial FMD lesions. **a** Aorta angiogram showing a long stenosis (*black arrow*) of the right renal artery, in favour of perimedial FMD. **b** Colour histological slide of perimedial FMD showing extensive perimedial fibrosis

This classification is not frequently used in daily practice because several types may co-exist in the same patient and/or the same artery. Today, pathological samples are analysed in exceptional circumstances. Kincaid et al.’s angiographic classification based on a series of 125 patients [21] is more commonly used. The purpose of Kincaid’s classification is to distinguish the multifocal (multiple stenosis or string-of-beads aspect), unifocal (short stenosis less than 1 cm long), tubular (stenosis more than 1 cm long) and mixed types. The string-of-beads aspect is the most characteristic aspect of FMD and indicates the presence of medial lesions [1]. The other angiographic aspects are less specific to a histological type.

In 2012, Savard et al. [22] proposed a simplified classification based only on two angiographic cervico-encephalic subtypes of FMD: the multifocal (presence of ≥2 stenoses on a given vessel segment with or without the typical string-of-beads appearance) and unifocal (presence of a single focal or tubular stenosis) types. Interestingly, Savard et al. showed significant clinical differences depending on the subtype such as median age at diagnosis of FMD, hypertension, sex distribution, initial blood pressure and current smoking.

#### Diagnostic tools

**Fig. 4:**
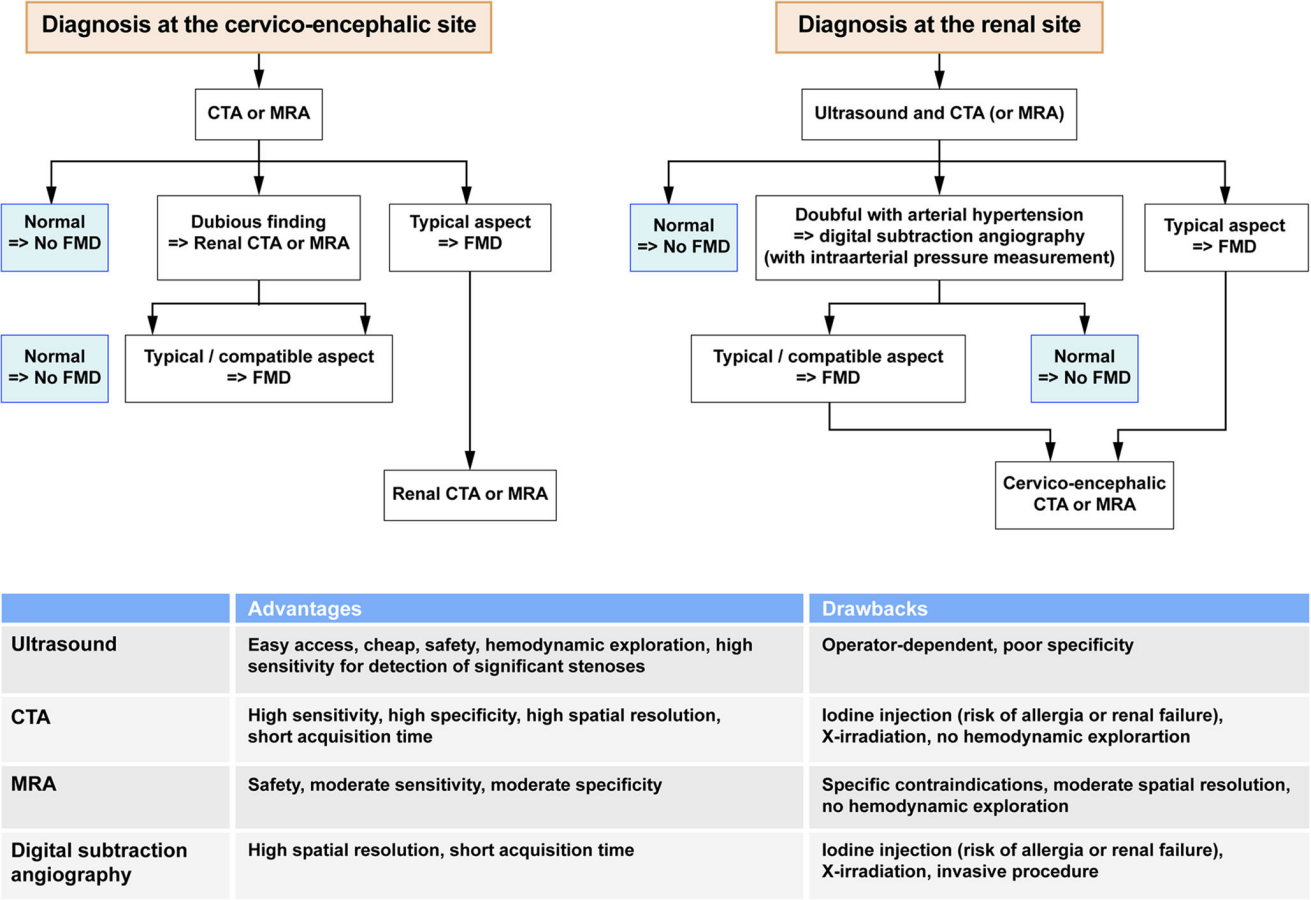
Flow chart illustrating imaging algorithms in case of suspected DFM for both the renal or cervico-encephalic level. The table shows the advantages and drawbacks of the different diagnostic tests

Figure 4 shows the diagnostic strategies, advantages and drawbacks of different imaging techniques.

**Fig. 5:**
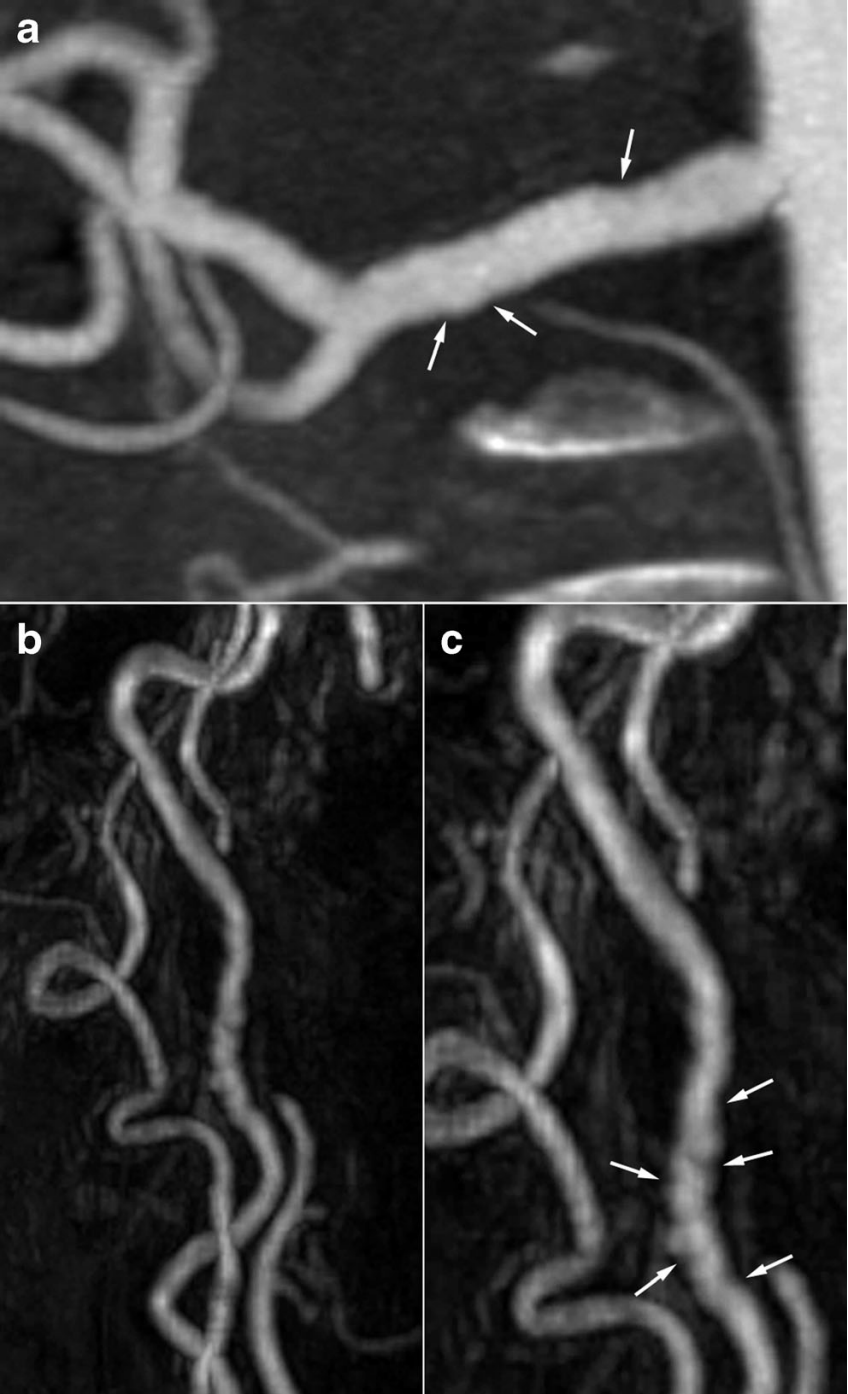
Radiologic findings in a 68-year-old female patient who suffered from an inferior myocardial infarct secondary to spontaneous dissection of the right coronary artery. **a** Renal coronal CTA MIP reconstruction showing moderate involvement of the right renal artery (*white arrows*). **b** and **c** Three-dimensional gadolinium-enhanced MRA reconstructions in the same patient showing the typical string-of-beads aspect (*white arrows*) of the internal carotid artery, highly suggestive of FMD

In case of renal impairment, Doppler ultrasound shows an accelerated blood flow in renal arteries with stenoses. It also allows studying the size of the kidneys, which is a good evaluation criterion for disease severity and follow-up [17].

Magnetic resonance angiography (MRA) and CTA present high specifities for detecting renal artery stenosis due to FMD (92 and 84 % respectively), but have relatively low sensitivities (64 and 62 % respectively) [1]. Although, MRA has a poorer spatial resolution (1–2 mm) compared with CTA, its main advantage is still the non-ionising technique. Finally, although gadolinium-based contrast material is still safer than iodine-based contrast media, gadolinium-based contrast agents should not to be used in patients with an estimated glomerular filtration rate <30 ml/min/1.73 m² because of concerns about nephrogenic systemic fibrosis [6]. In these patients, non-contrast MR angiography techniques with time-of-flight sequences can be used especially in ultra-high field MRI equipment. Nonetheless, to date, no published studies have validated the use of time-of-flight sequences for the diagnosis of FMD compared with angiography or other non-invasive imaging modalities [23]. A recent study comparing contrast-enhanced MRA with conventional angiography showed a sensitivity of 97 % and specificity of 93 % for FMD diagnosis. MRA was more sensitive for detecting the string-of-beads appearance (97 %) than detecting a >50 % stenosis (68 %) [24].

CTA has the best sensitivity/specificity ratio (1) and allows reconstruction of the renal arterial vasculature [23] but requires contrast media injection. High spatial resolution and short acquisition time are the major advantages of the current CTA studies. This technology allows visualisation of a greater volume per unit time, resulting in reduced pulsation and stair-step artefacts. CTA has the ability to generate three-dimensional multiplanar and volume-rendered images. Assessment of the renal arteries should be performed using vascular windows on a dedicated three-dimensional workstation. It is imperative to review the data sets using multiple reconstruction formats, including multiplanar reformatted images, shaded surface display and maximum-intensity projections. The use of all these reformats in addition to the axial “raw data” has been shown to improve the sensitivity and specificity of this imaging modality [25].

Although the diagnosis of cervico-encephalic FMD can be suspected with Doppler ultrasound in case of vascular loops, ectasia, intimal flap or aliasing, it is most frequently established with cross-sectional imaging, using CTA of the supra-aortic trunks (SAT), MRA or in some cases conventional angiography. These imaging techniques present better sensitivity in detecting lesions of the middle and distal portions of the internal carotid and vertebral arteries at the level of the C1 and C2 vertebrae [1], which are the most frequently affected segments [3].

Multi-row detector CTA allows a detailed evaluation of the extracranial and intracranial cerebrovasculature with the ability to identify FMD, dissections, cerebral aneurysms and atherosclerosis. Moreover, the images can be reconstructed in maximal intensity and three-dimensional projections, allowing detailed anatomic visualisation.

No clinical studies have validated the use of MRA compared with catheter-based angiography for the diagnosis of cerebrovascular FMD. Consequently, the specificity and sensitivity of MRA in the diagnosis of FMD are not known. Nevertheless, MRA may have the benefit of detecting FMD-associated SAT dissections when T1 fat-saturation images are acquired simultaneously with time-of-flight or gadolinium-enhanced images [26].

Despite the improvements in non-invasive screening techniques, negative results do not exclude the diagnosis of FMD. In case of high clinical suspicion, use of diagnostic arteriography [1, 9] or endovascular ultrasound can be discussed if therapeutically relevant. Indeed, catheter-based angiography remains the gold standard imaging modality for renovascular FMD because of its unsurpassed spatial resolution (<0.1 mm) [1, 3].

##### Imaging acquisition protocols

To date there are no existing imaging protocol recommendations specific to FMD diagnosis. In our institution, the following protocols are currently used for both the initial diagnosis and follow-up:

- Renal
  - CTA

An ROI is placed in the abdominal aorta; density tracking is used with a threshold set at 180 HU and 3-s delay. Seventy ml of Omnipaque 350 is used. ANgioCT is performed from the diaphragm to pubis. The slice thickness is 1 mm, with the following settings: 120 kV, 300 mAs.

- MRA

This protocol can be randomly used in both 1.5- and 3-T scanners: Coronal 3D FFE angioMR after injection of 0.2 ml/kg of Dotarem at 2 ml/s. Slice thickness is set at 0.8 mm (total slices 65), TR: 5 ms, TE: 1,79 ms.

- SAT
  - CTA

An ROI is set in the aortic arch with a 75-DH threshold with no delay. Acquisition is set from the tracheal bifurcation to 4 cm above the dorsum salae. Injection of 60 ml of Omnipaque 350 is at 5 cc/s. Slice thickness is 0.9 mm with 240 mAs, 120 kV.

- MRA

Coronal 3D FFE angioMR is carried out after injection of 0.2 ml/kg of Dotarem at 1.2 ml/s. Slice thickness is set at 0.7 mm (total slices 105) with the following settings: TR: 4 ms, TE: 1.57 ms.

#### Preferential locations, clinical manifestation and radiological diagnostic criteria

##### 1. Renal arteries

The renal arteries are the most frequently affected location of FMD lesions. In 1997, in a series of 104 hypertensive patients with renal artery FMD, 80 % were middle-aged females with preserved renal function. In 54 % of the cases, the lesions were bilateral, and they involved the branches in 42 % of the cases. Multifocal string-of-beads lesions were found in 84 % of the cases. The unifocal type most often affected young males, with tighter stenosis and more frequent downstream lesions [27].

FMD renal involvement does not necessarily lead to arterial hypertension, and progression toward kidney failure is rare, even in cases of bilateral lesions [1, 12]. In case of stenosis in a patient with hypertension and renal FMD, the severity of stenosis and its relation to hypertension can only be asserted in case of asymmetrical kidney size. Measuring intra-renal arterial pressure compared to intra-aortic pressure can also assess the severity of stenosis. Thus, in case of a significant difference (>20 mmHg), hypertension can be attributed to the FMD lesions and therefore lead to renal angioplasty.

Even in cases of non-significant renal artery stenosis, the progression of the lesions ought to be monitored. In nearly one-third of cases, occurrence of a new lesion, worsening stenosis or an extending aneurysm can be visualised in these patients [21].

##### 2. Cervico-encephalic arteries

The advent of multi-detector CT and the increase in awareness among physicians about the cervico-encephalic location of FMD have certainly participated in increasing the its prevalence. In 1982, Mettinger and Ericson, from an analysis of the literature about FMD, reported a prevalence of cervico-encephalic FMD lesions of 32 % (in about 1,100 patients) [9]. In 2012, Olin et al. [6] reported a much higher prevalence of 74 % (in 447 cases from the FMD registry from nine states in the USA).

Although the most frequent symptoms of FMD are non-specific (headache, dizziness, neck pain) [6], some clinical manifestations of cervico-encephalic FMD are more serious, such as transient ischaemic attack (TIA), stroke, subarachnoid haemorrhage and arterial dissection. FMD mainly affects the internal carotid artery in its extracranial part, but all territories can be affected [17].

TIA and ischaemic stroke can be due to either arterial stenosis, leading to hypoperfusion and clot emboli, or to spontaneous extracranial arterial dissection. In case of intracranial dissection, haemorrhage most often occurs. FMD can also lead to subarachnoid haemorrhage (SAH) because of the occurrence of an intracranial dissection or a rupture of intracranial aneurysms (mainly due to the rupture of the internal elastic lamina in the medial types of FMD) [17].

##### 3. Radiological diagnostic criteria

There are no formal radiological diagnostic criteria, but it is well established that the string-of-beads aspect found in renal or cervico-encephalic locations on arteriography, CTA or MRA is highly suggestive of FMD. Focal or tubular lesions on angiography allow determining the diagnosis when they are typical [28]. Another frequent finding suggestive of an FMD diagnosis is the presence of a “web-like” defect at the origin of the internal carotid artery [3, 29].

**Fig. 6:**
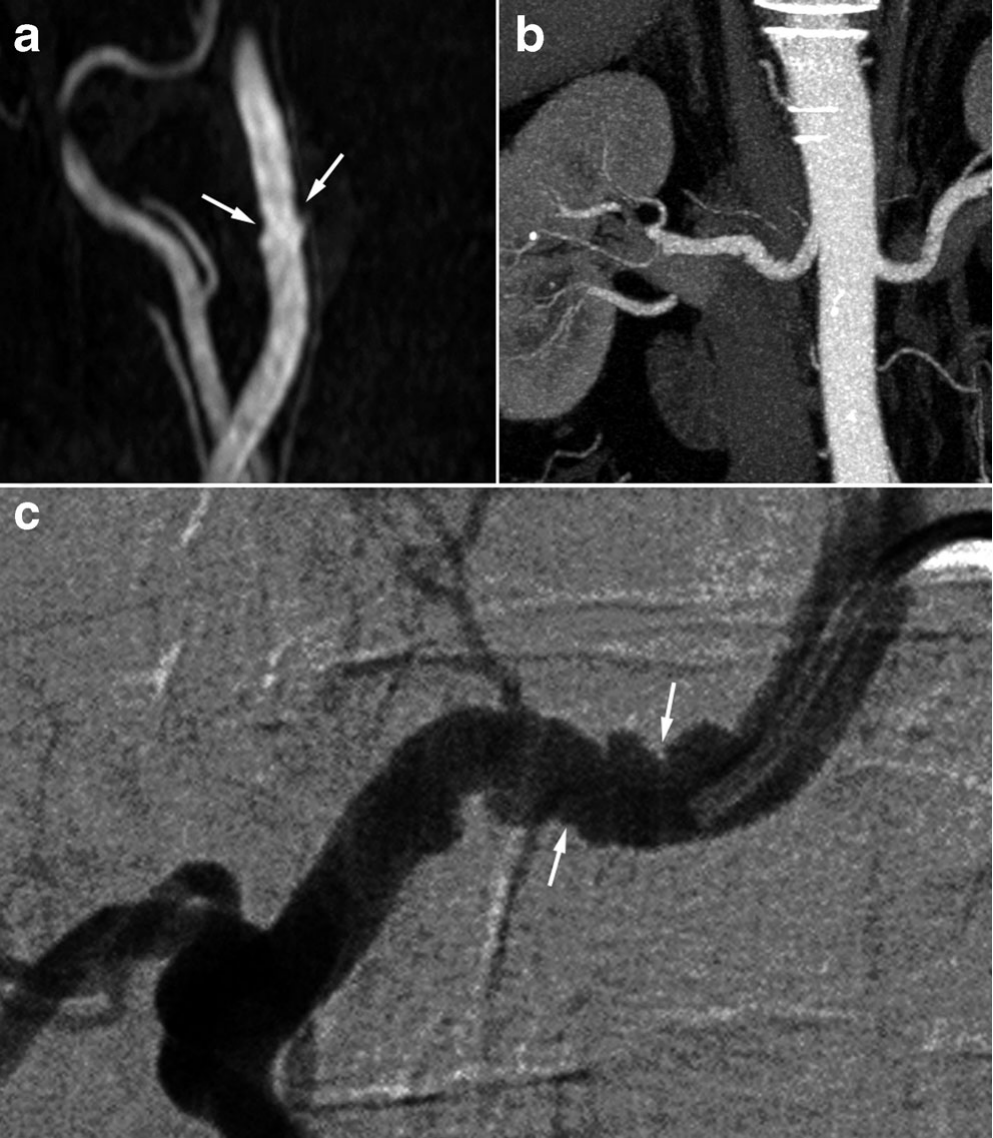
Radiologic findings in a 62-year-old male patient with arterial hypertension. **a** Gadolinium-enhanced MRA with MIP reconstruction showing typical but discrete involvement of the internal carotid artery (*white arrows*). **b** Renal CTA, coronal MIP reconstruction in the same patient showing a typical aspect of a medial string-of-beads dysplasia of the right renal artery. **c** Right renal angiogram confirming the typical aspect of the right renal artery, in favour of FMD

In such situations, lesions may be discrete at the cervical level and typical at the renal level. Thus, imaging of the renal arteries will help to establish the diagnosis. The opposite situation is also true, and performing imaging of the renal arteries is recommended in a patient presenting discrete but suggestive lesions in the cervico-encephalic arteries so as to confirm the diagnosis, and vice versa (Figs. 5 and 6) [30].

**Fig. 7:**
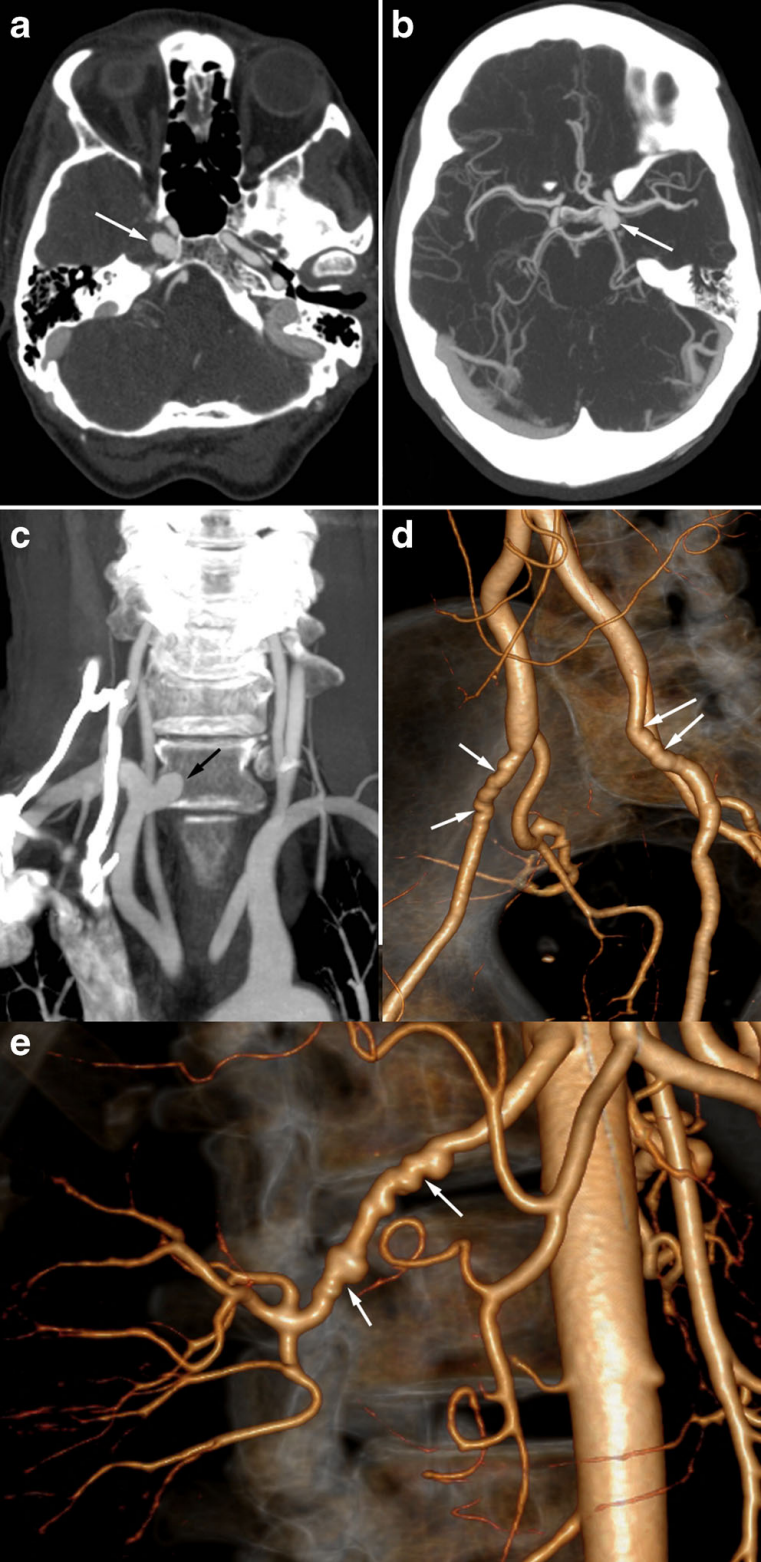
Radiologic findings in a 56-year-old female patient presenting with non-specific headaches. **a** and **b** Cerebral CTA showing bilateral carotid aneurysms with intracavernous development (*white arrows*). **c** Coronal CTA MIP reconstruction of the supra-aortic trunks, showing a right subclavicular aneurysm (*black arrow*). **d** and **e** Three-dimensional volume-rendered abdominal CTA showing typical medial FMD affecting the external iliac arteries (*white arrows*, **d**) and the right renal artery (*white arrows*, **e**)

Intracranial FMD with a typical string-of-beads aspect (basilar artery, carotid, middle cerebral artery) is usually an intracranial extension of extracranial lesions [29].

### Less typical presentations (Fig. 12)

#### Less typical locations

FMD can occur in almost any artery, including those that supply the intestines (mesenteric arteries) and the upper (brachial arteries) and lower limbs (iliac arteries). In many patients, FMD is found in more than one artery, which argues for a systemic disease. In 2012, Olin et al. [6] reported that FMD affects mesenteric arteries in 26.3 %, upper extremity arteries in 15.9 % and lower extremity arteries in 6 %. No aortic location was reported in this study. To date, it is not possible to determine whether the coronary artery involvement is due to atherosclerosis or fibromuscular dysplasia.

#### Arterial dissection

According to Olin et al., out of 447 cases of FMD, 19.7 % had an arterial dissection [6]. In 75 % of cases, the dissection was located in the carotid artery, 22 % in the renal artery and 17 % in a vertebral artery.

Renal artery dissection may occur in 5–10 %, especially in case of renal artery tubular stenosis [31]. It may be responsible of renal infarct by total occlusion or distal emboli, leading to sudden pain in the flank, haematuria and/or rapidly progressive hypertension [1].

Extracranial dissection of the neck arteries, symptomatic or not, may occur as a complication of FMD lesions [4, 32]. However, it is not sufficient to establish a final diagnosis of FMD. In our experience, out of 64 patients with FMD, more than half of the cervico-encephalic artery dissections were discovered unexpectedly (unpublished data). Spontaneous dissection of a cervical artery is a frequent cause of stroke in young adults and may be associated with FMD in 15–20 % of cases [33, 34]. For this reason, physicians should consider FMD in patients with cervico-encephalic artery dissection, particularly if it is spontaneous, multifocal or in an atypical location. This situation should lead to further exploration of the renal arteries. The same approach could be proposed in case of a pseudoaneurysm that might correspond to the aftermath of a dissection.

#### Aneurysms/vascular ectasia

Single or multiple aneurysms are frequent in FMD patients [5, 35]. Their prevalence reached 17 % in Olin et al.’s series [6]. Their preferential locations are the renal arteries (33 %) where large aneurysms may develop, notably in medial FMD. Their rupture remains a rare complication. They may also be responsible for distal emboli or even arteriovenous fistula when rupturing in the renal vein [36]. The most frequent locations are the following: carotid (21 %), aortic (19.7 %) and coeliac (15.8 %). A strong association also exists between FMD and intracranial aneurysm, accounting for 11.8 % of the aneurysms in the series reported by Olin et al. Several authors report a 22–51 % prevalence of intracranial aneurysms in patients with FMD in the carotid and/or vertebral arteries [9, 29, 37, 38]. Nonetheless, these results come from a series of cerebral angiograms for the most part undertaken for hypertension. In 1998, in a meta-analysis of 18 studies including 615 FMD patients, Cloft et al. found a 24 % prevalence of intracranial aneurysms [39]. However, in the 212 patients without SAH, the prevalence was 7.3 %, which remains higher than the prevalence expected in the general population, around 2.3 % [40]. One may here suggest the diagnosis of FMD in patients with intracranial aneurysm and/or carotid ectasia, particularly in young females, especially in cases of an associated personal or familial history of hypertension.

In some cases, these aneurysms have an intracavernous development and may be bilateral (Fig. 7). They therefore do lead to SAH but can be responsible for cavernous sinus syndrome. An exceptional case of a carotid-cavernous fistula has even been described [35]. Loops and/or fusiform vascular ectasia in the subpetrous segment of the internal carotid arteries is also frequently found in FMD patients [30].

**Fig. 8:**
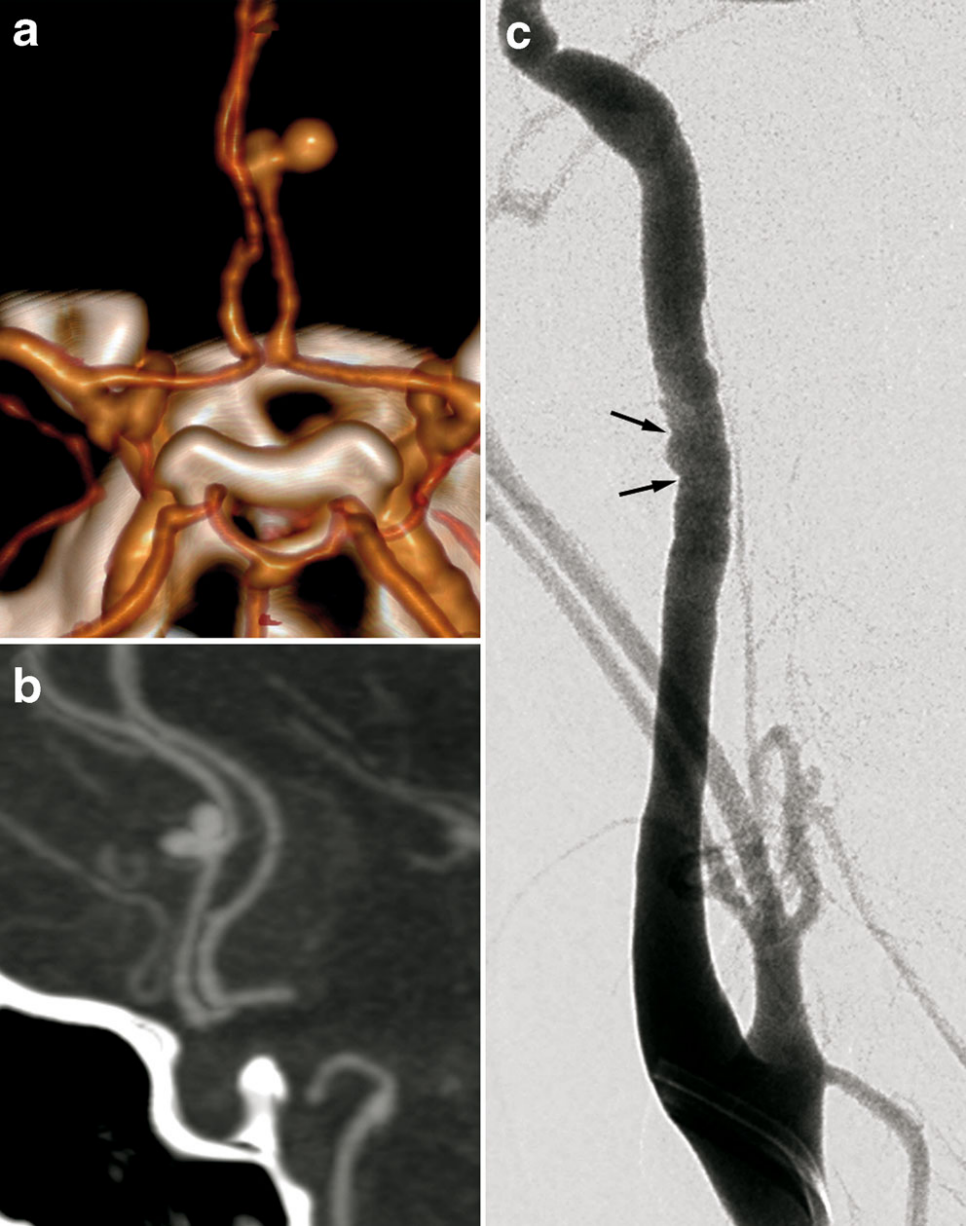
Radiologic findings in a 38-year-old female patient presenting with non-traumatic spontaneous subarachnoid haemorrhage. **a** Three-dimensional volume-rendered cerebral CTA showing a right peri-callosal artery aneurysm. **b** Cerebral CTA sagittal MIP reconstruction of the same aneurysm. **c** Therapeutic arteriography revealed typical right carotid artery medial FMD lesions (*black arrows*)

#### Unexplained SAH

**Fig. 9:**
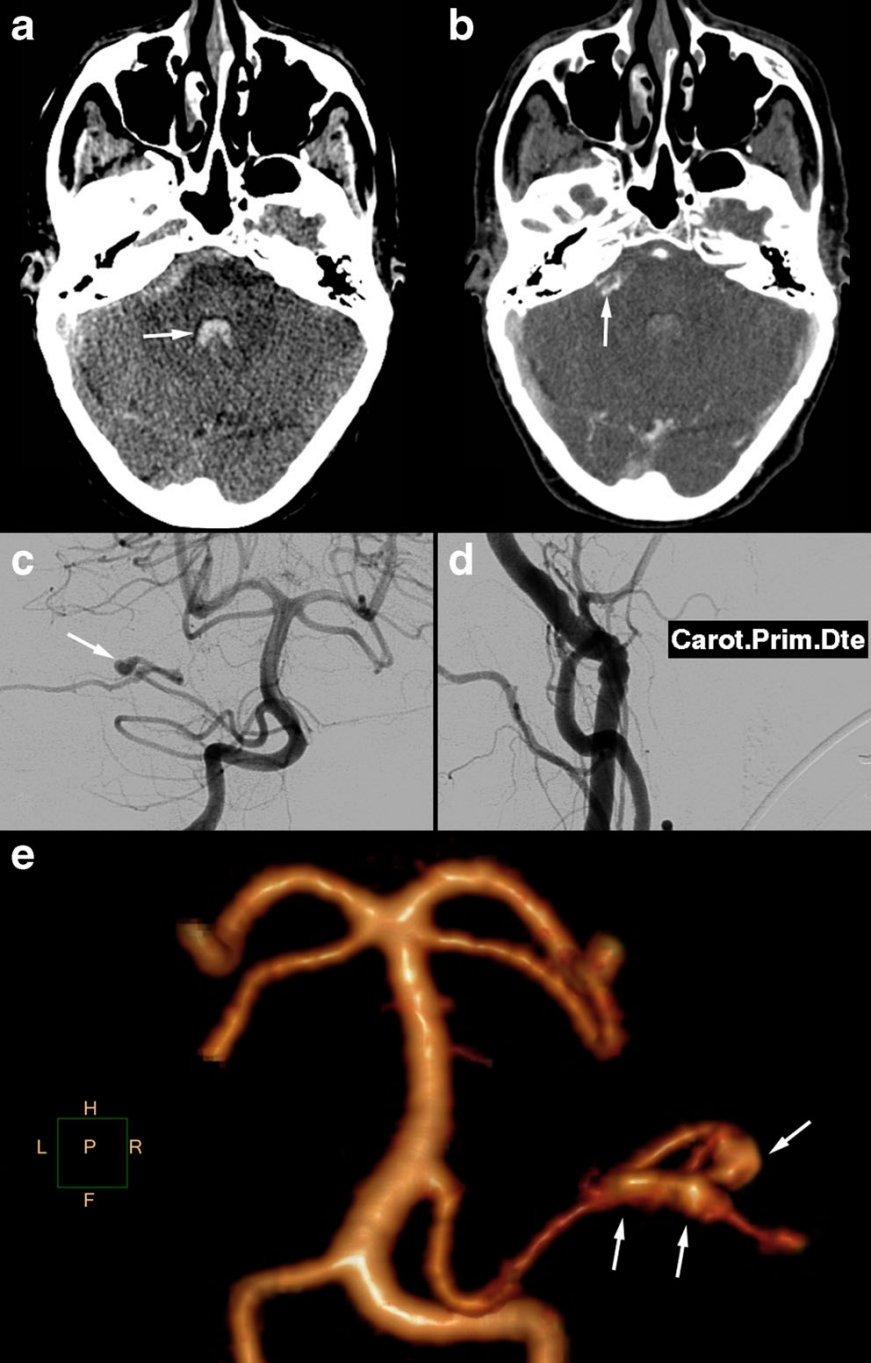
Radiologic findings in a 70-year-old female patient presenting a right-hemisphere non-traumatic SAH with tetraventricular inundation. **a** Cerebral non-enhanced CT showing SAH and intraventricular haemorrhage. **b** Cerebral CTA showing a dissecting aneurysm of the right antero-inferior cerebellar artery. **c** Angiogram of the right vertebral artery in arterial phase confirming the dissecting aneurysm of the right antero-inferior cerebellar artery (*white arrow*). **d** Right common carotid angiogram showing the typical string-of-beads aspect, in favour of FMD. **e** Cerebral 3D volume-rendered CTA of the dissecting aneurysm of the right antero-inferior cerebellar artery (*white arrows*)

**Fig. 10:**
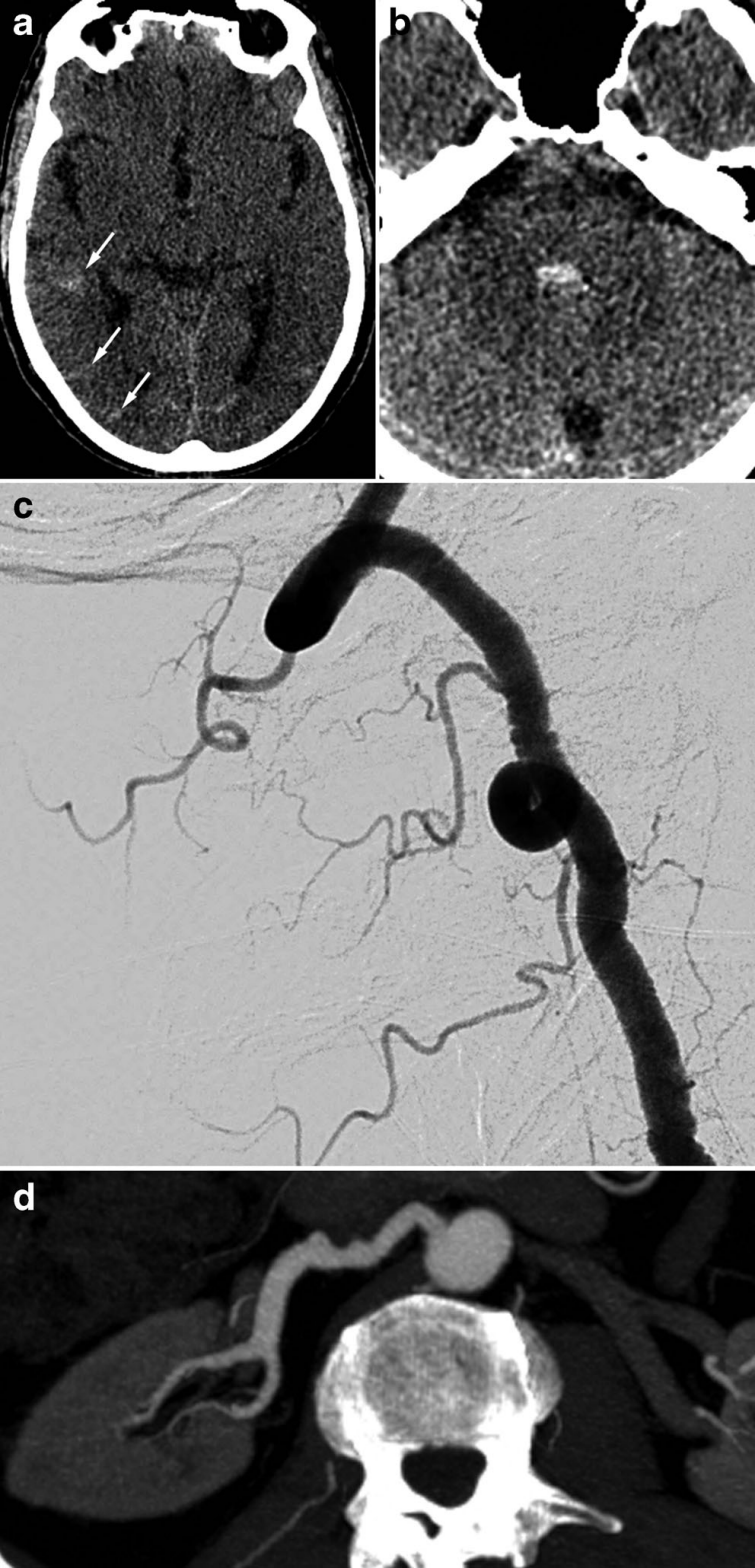
Radiologic findings in a 65-year-old male patient with SAH of the posterior fossa. The CTA shows no particular signs. The arteriography found typical FMD lesions in the right vertebral artery. The renal CT shows ectasia of the right renal artery termination. **a** and **b** Cerebral non-enhanced CT showing right temporo-occipital SAH (*white arrows*) associated with intraventricular haemorrhage (V4). **c** Right vertebral angiogram showing the typical string-of-beads aspect. **d** Renal CTA, curved reconstructions of the right renal artery showing ectasia of the distal part of the right renal artery

**Fig. 11:**
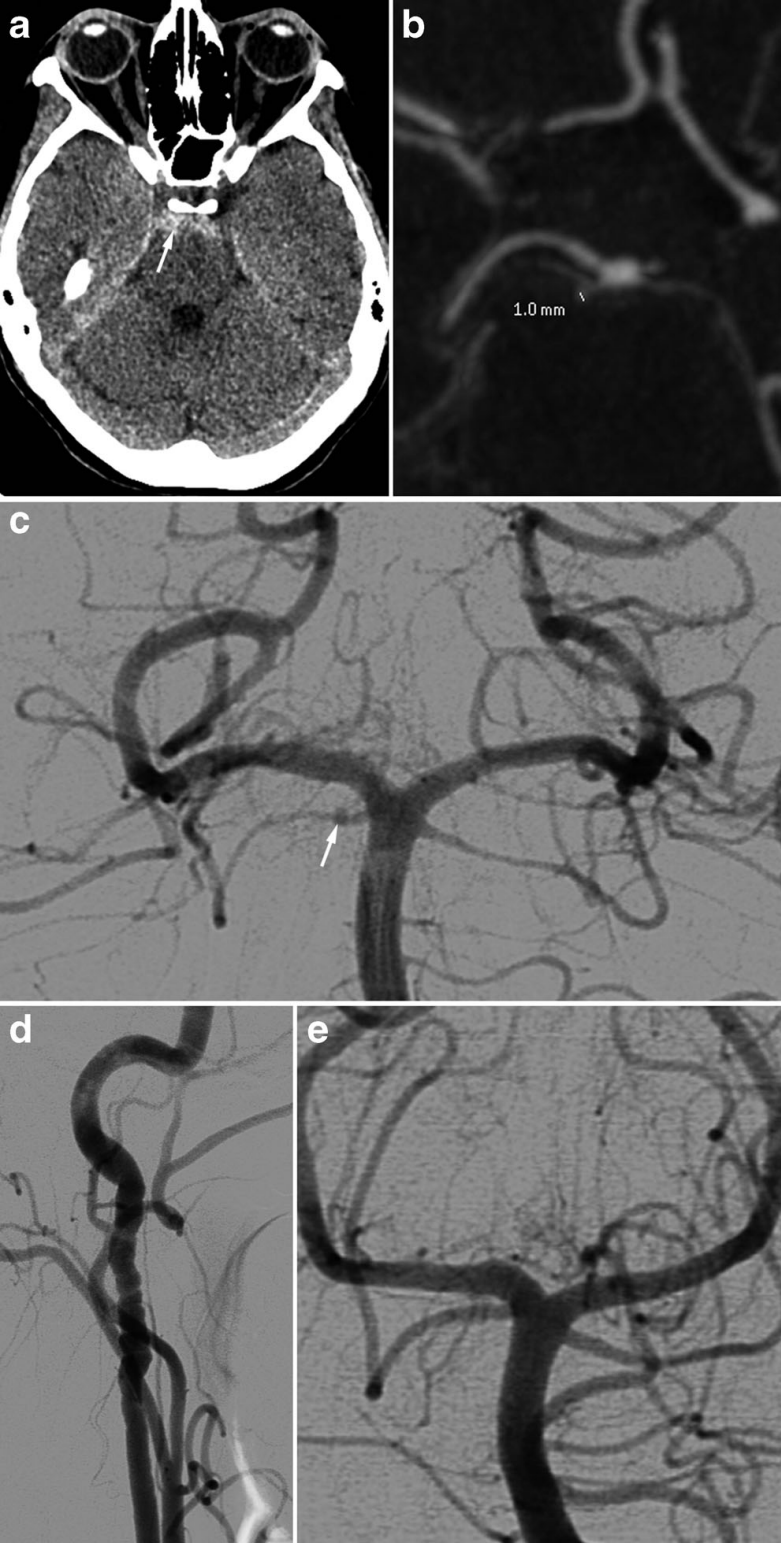
Radiologic findings in a 48-year-old female presenting with posterior headaches. The initial CT shows SAH of the posterior fossa. The angiography found dissection of the right superior cerebellar artery. Study of the supra-aortic arteries shows typical FMD images. Ad integrum restitution at 3 months. **a** Cerebral non-enhanced CT showing prepontine SAH (*white arrow*). **b** and **c** Cerebral CTA and angiogram showing a dissection of the right superior cerebellar artery. **d** Right carotid angiogram showing showing classical FMD imaging findings. **e** The 3-month follow-up angiography showing ad integrum restitution of the artery, in favour of arterial dissection

**Fig. 12:**
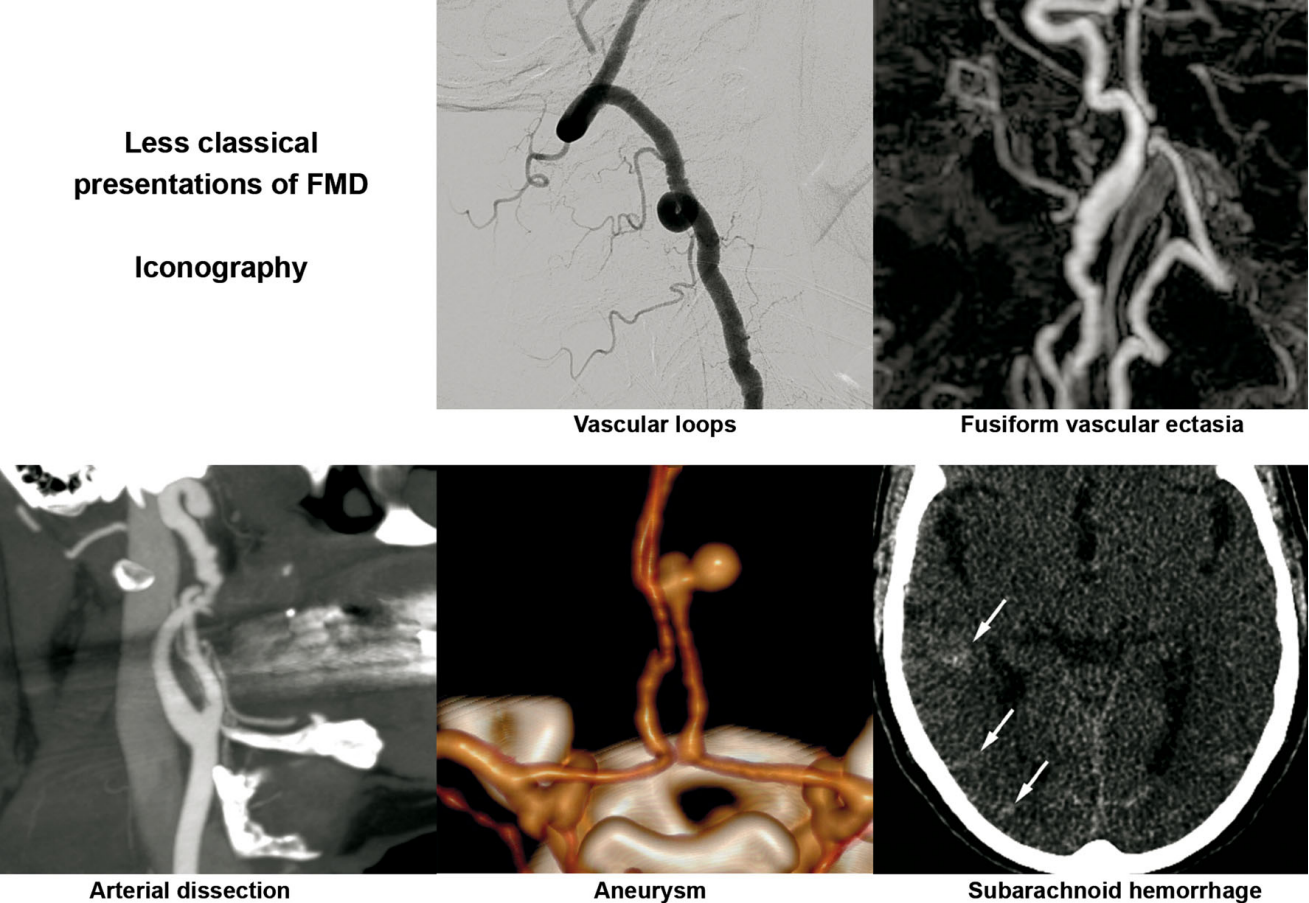
Summary of less typical imaging findings in FMD

SAH found in FMD patients is usually a consequence of subarachnoid intracranial aneurysm rupture (Figs. 8 and 9) [3]. SAH has also been described in patients with no visible aneurysm on angiography but with ruptured of microaneurysms of the basilar artery at autopsy (Fig. 10) [41]. These SAHs can also stem from intracranial dissection [30, 42]. The diagnosis is suggested on arteriography (Figs. 4, 11 and 12). For intracranial dissection with SAH re-bleeds, usually endovascular or neurosurgical treatment can be proposed. In the case the arteriography at 3 months showed an ad integrum restitution of the artery.

Thus, the presence of non-traumatic SAH, especially in a young female patient, wherever it may be located and whatever its size, should bring up the diagnosis and discussion of a cervico-encephalic artery and renal artery examination during the diagnostic work-up [30].

## Differential diagnoses (Fig. 13)

**Fig. 13:**
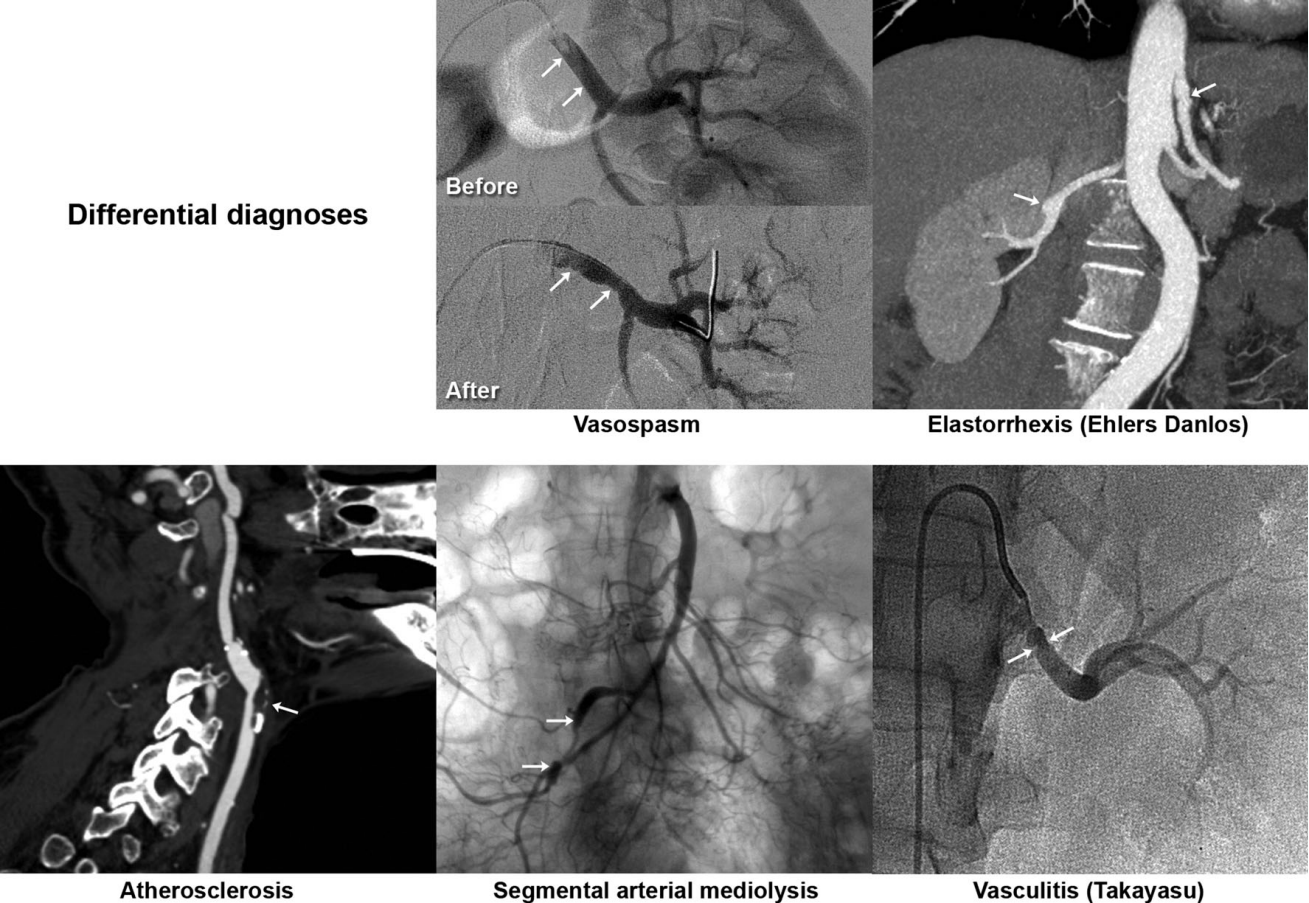
Summary of classical differential diagnoses

First of all, one must ensure that the images do not stem from vasospasm or stationary waves related to catheter use and/or the injection of a contrast agent. This is manifested by regular undulations with no significant stenosis, which are reversible after injection of a vasodilator and/or removal of the catheter [43].

Marfan or vascular Ehlers-Danlos syndrome should be discussed when presented with aneurysmal lesions and/or multiple dissections, particularly with a compatible morphotype or in a suggestive family context [1]. These diagnoses rely on associated phenotypic traits and genetic tests: acrogeric dysmorhy, distal joint laxity and tiny skin elasticity (confirmed by detection of the COL3A1 gene mutation) [44].

Vasculitis may mimic FMD lesions, but in most cases the presence of systemic inflammation provides the diagnosis [1].

Atherosclerotic lesions usually occur in a different setting. Most focal lesions are preferentially located at the ostium of the arteries or at a bifurcation. Most FMD patients are young and have few or no cardiovascular risk factors or aortic atheromatous plaques [1].

Finally, segmental arterial mediolysis (SAM) should be suggested in cases of an association of digestive tract artery lesions, notably the gastroduodenal artery. The similarity between the chronic vacular lesions of SAM and FMD lesions raises the question of a common pathophysiological stem of these lesions, one perhaps being a progressive form of the other [45, 46].

With typical string-of-beads lesions involving the renal and/or carotid arteries, possible differential diagnoses are limited.

## Treatment/management

Treatment for patients with FMD may include medical therapy and surveillance; endovascular therapy for stenosis (angioplasty with or without stenting), dissection (stents) or aneurysms (coils, stents); or surgery. Therapeutic decisions depend on the nature and location of vascular lesions (stenosis versus dissection versus aneurysm), the presence and severity of symptoms, prior to vascular events related to FMD, the presence and size of aneurysms, and comorbid conditions.

The lack of knowledge about the natural history of FMD and the insufficient number of randomised studies comparing different therapeutic approaches does not allow adequate evaluation of the effectiveness of different therapeutic options. In patients who had an ischaemic stroke antiplatelet therapy is generally introduced, although its efficacy has never been demonstrated specifically for symptomatic patients with FMD.

Patients with symptomatic FMD lesions that are accessible to surgery and with low perioperative risk can be good candidates for surgery because the long-term anatomical results are good and most surgical techniques are well known.

However, because of recent advances in interventional radiology techniques, including angioplasty with or without stenting showed, patients treated percutaneously have fewer complications. This is why patients who present recurring symptoms resulting from haemodynamic instability, despite medical treatment, may be offered percutaneous angioplasty as it is considered the most reasonable treatment option. However, the results of long-term percutaneous angioplasty are still poorly understood [3].

## Conclusion

FMD is a systemic non-atheromatous, non-inflammatory disease that affects females more than males. Renal and cervico-encephalic arteries are the most frequently affected sites. However, FMD may affect any artery. Radiologists play an important role in the diagnosis of FMD. A better knowledge of this disease is critical to shorten the delay between the first symptoms and final diagnosis. Common imaging findings of FMD, mainly the usual string-of-beads aspect, are well known. However, it is important to recognise less common presentations of FMD, such as vascular loops, fusiform vascular ectasia, arterial dissection, aneurysm and subarachnoid haemorrhage, to suggest the diagnosis and to conduct further investigations.
